# Pan-caspase inhibition during normothermic machine perfusion of discarded livers mitigates *ex situ* innate immune responses

**DOI:** 10.3389/fimmu.2022.940094

**Published:** 2022-07-26

**Authors:** Siavash Raigani, John Santiago, Anders Ohman, Megan Heaney, Sofia Baptista, Taylor M. Coe, Reinier J. de Vries, Ivy Rosales, Angela Shih, James F. Markmann, Philip Gruppuso, Korkut Uygun, Jennifer Sanders, Heidi Yeh

**Affiliations:** ^1^ Division of Transplant Surgery, Massachusetts General Hospital, Boston, MA, United States; ^2^ Center for Engineering in Medicine and Surgery, Massachusetts General Hospital and Harvard Medical School, Boston, MA, United States; ^3^ Department of Pediatrics, Rhode Island Hospital and Brown University, Providence, RI, United States; ^4^ Department of Pathology, Massachusetts General Hospital, Boston, MA, United States

**Keywords:** machine perfusion, liver transplant, apoptosis, caspase, normothermic, ischemia reperfusion injury

## Abstract

Access to liver transplantation is limited by a significant organ shortage. The recent introduction of machine perfusion technology allows surgeons to monitor and assess ex situ liver function prior to transplantation. However, many donated organs are of inadequate quality for transplant, though opportunities exist to rehabilitate organ function with adjunct therapeutics during normothermic machine perfusion. In this preclinical study, we targeted the apoptosis pathway as a potential method of improving hepatocellular function. Treatment of discarded human livers during normothermic perfusion with an irreversible pan-caspase inhibitor, emricasan, resulted in significant mitigation of innate immune and pro-inflammatory responses at both the transcriptional and protein level. This was evidenced by significantly decreased circulating levels of the pro-inflammatory cytokines, interleukin-6, interleukin-8, and interferon-gamma, compared to control livers. Compared to emricasan-treated livers, untreated livers demonstrated transcriptional changes notable for enrichment in pathways involved in innate immunity, leukocyte migration, and cytokine-mediated signaling. Targeting of unregulated apoptosis may represent a viable therapeutic intervention for immunomodulation during machine perfusion.

## Introduction

Liver transplantation (LT) provides the only definitive cure for end-stage liver disease, though access is limited due to a shortage of donor organs. The growing use of *ex situ* machine perfusion technology for dynamic liver preservation prior to LT has significantly expanded the use of marginal and extended-criteria grafts in recent years (1, 2). However, sizeable knowledge gaps remain with respect to liver physiology during normothermic machine perfusion (NMP) that limit its potential use as a platform for rehabilitation of untransplantable or severely injured grafts. Recent work from our collaborative group has delineated differences in liver physiology during NMP relative to hepatocellular function as defined by the balance between ischemia-reperfusion injury (IRI) and recovery of cellular homeostasis *via* autophagy (3). Therefore, leveraging the equilibrium between graft injury and recovery with adjunct therapeutic interventions during NMP may present a reliable avenue for rehabilitation of graft function and transplantation of livers with inadequate function.

One approach to “tipping the scale” between IRI and autophagy may be to target cell death pathways that represent the consequence of irrecoverable injury (4, 5). Apoptosis, pyroptosis, and necroptosis represent different forms of tightly controlled stress-induced regulated cell death mechanisms in the liver (6). Under normal physiologic conditions, apoptosis allows for controlled removal of injured liver cells followed by cellular proliferation of surrounding healthy hepatocytes. Autophagy is another homeostatic control mechanism in which damaged organelles and proteins are degraded, thus protecting cells from undergoing apoptosis. Crosstalk between apoptosis and autophagy can dictate a cell’s fate (5). However, under abnormal conditions, apoptosis can be pathologic if the equilibrium between turnover and proliferation is disrupted, resulting in chronic inflammation, fibrosis, and even cancer (7). Dysregulated apoptosis has been described in the setting of liver disease and transplant for decades and numerous studies have shown that therapeutic inhibition of apoptosis results in mitigation of IRI (8–13).

Given that our previous work demonstrated the association of hepatocellular function during NMP with the balance between liver injury and autophagy, we hypothesized that discarded human livers with inadequate hepatocellular function during NMP would be characterized by an insufficient autophagic response with resultant overwhelming apoptosis. We subsequently tested whether adjunct delivery of an irreversible pan-caspase inhibitor, emricasan, during NMP would improve hepatocellular function of discarded human livers by shifting the equilibrium between cell death and autophagy.

## Materials and methods

### Human liver perfusions

Sixteen human livers from donation after circulatory death, with consent for research, were included in this study after being turned down by all transplant centers in the respective region of procurement. Eleven livers had been previously described in a research cohort of machine perfused discarded human livers (14) and comprised the control groups. Additional details regarding procurement, enrollment, and bench preparation are provided in the supplemental methods. Donor risk index (DRI) was calculated according to Feng et al. (15).

Grafts were perfused on the Liver Assist device (Organ Assist, Groningen, Netherlands) using a previously described protocol (14). Briefly, perfusate composition consisted of O+ packed red blood cells, human albumin, Lactated Ringer’s solution, and heparin. Bile salts (taurocholate) and lipid-free parenteral nutrition were continuously infused. Perfusate, bile, and tissue biopsies were collected and analyzed at multiple time points. Grafts were assessed for adequate hepatocellular and cholangiocellular function. Hepatocellular function was defined as the ability to clear lactate below a threshold of 2.5 mmol/L, in addition to demonstrating stable vascular flow and pH without sustained bicarbonate supplementation. Cholangiocellular (biliary) viability metrics were measured according to van Leeuwen (16).

**Figure 1:**
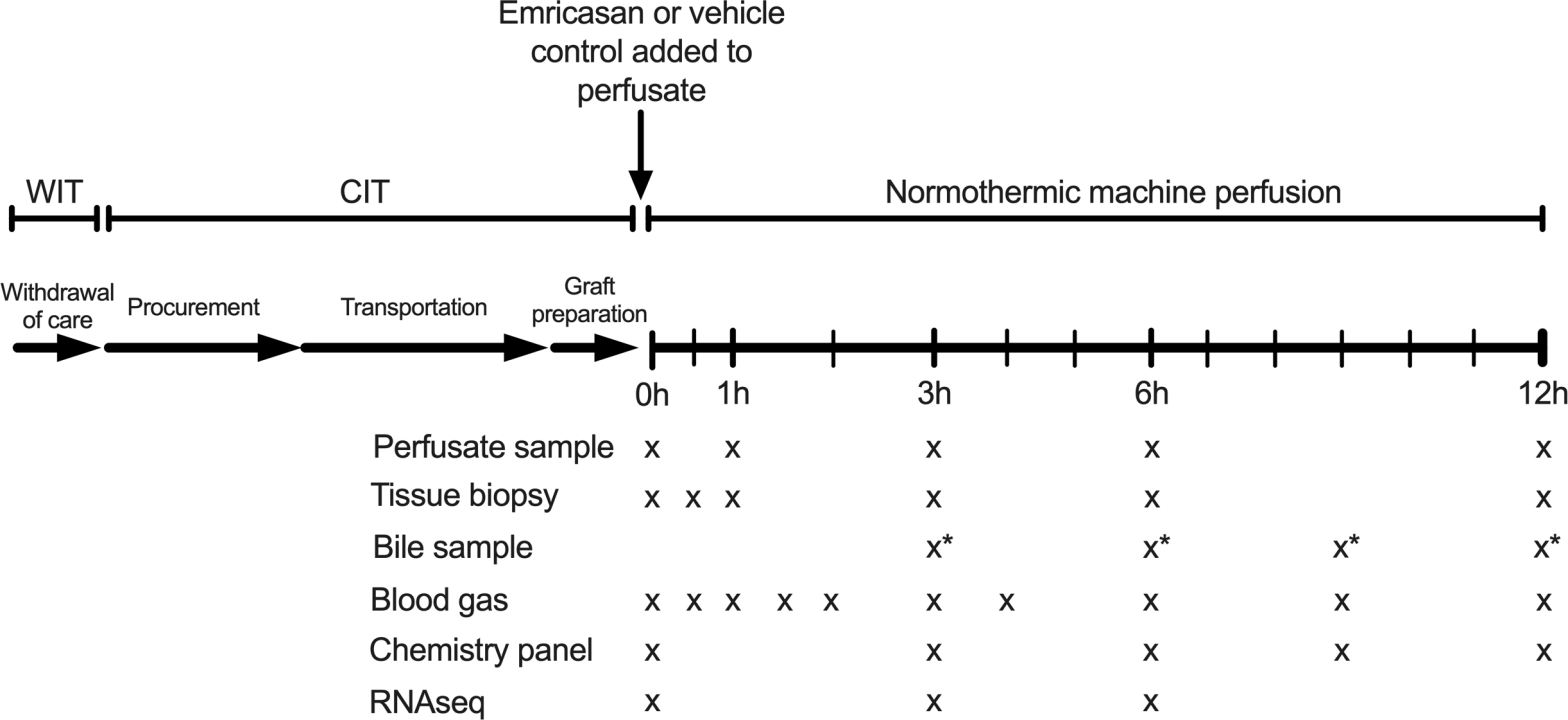
Liver preservation and NMP protocol. Emricasan (5mg/kg liver) or vehicle control (1mL DMSO) was added to the circulating perfusate immediately prior to connection of the liver to the NMP device. Serial tissue biopsies and perfusate samples were taken at indicated time points for subsequent analysis. * indicates a discordance between EM perfusions (bile samples taken at 3, 6, 9, and 12 hours) and control perfusions (bile samples taken at 4, 8, and 12 hours). NMP, normothermic machine perfusion; WIT, warm ischemic time; CIT, cold ischemic time; RNAseq, bulk RNA sequencing.

Of the 11 livers comprising the control groups, six demonstrated adequate hepatocellular function (AHF) and five did not (inadequate hepatocellular function, IHF). With respect to the experimental group (emricasan, EM), five consecutive human livers turned down for transplant were enrolled. Emricasan, also known as IDN-6556 (MedChemExpress, Monmouth Junction, NJ), dissolved in DMSO was added to the circulating perfusate prior to initiation of liver perfusion at a dose of 5mg/kg liver weight (1mL total volume, 0.05% v/v total perfusate). Vehicle control (1mL DMSO) was previously incorporated in the control liver perfusions (**Figure 1**).

### RNA sequencing

Core needle biopsies taken immediately prior to perfusion and after 3 and 6 hours of perfusion were used for transcriptome sequencing. Methods of tissue collection, purification, and bioinformatic analysis have been previously described (3) and are detailed in the **Supplemental Methods**. The differential gene expression threshold for significance was set to a Benjamini-Hochberg false discovery rate (FDR) < 0.05. Raw sequence data have been deposited in the Gene Expression Omnibus with accession no. GSE202565 and no. GSE165568 (open access).

### Protein analysis

Perfusate collected at the indicated time points was centrifuged at 5000g and the plasma collected and stored at -80°C for later analysis. Enzyme-linked immunosorbent assays (ELISA) were performed for various proteins according to manufacturers’ guidelines (**Supplemental Methods**).

Sequential wedge biopsies from the right lobe of each liver were performed at indicated intervals, flash frozen in liquid nitrogen, and stored at -80°C. Tissue samples (20mg) were homogenized and Western immunoblots performed as previously described (17). A full list of antibodies used is provided in the **Supplemental Methods**.

### Histology and immunohistochemistry

Core needle biopsies taken from the right liver lobe at indicated intervals were fixed in formalin and embedded in paraffin. Hematoxylin and eosin (H&E) stains were performed on all biopsies to assess the degree of necrosis, inflammatory cell infiltrate, and reperfusion injury using a validated scoring system (18). Immunohistochemistry for LC3 was performed as previously described (3).

### Statistical analysis

Categorical data are presented as median with interquartile range (IQR) and frequency data as percentages. For statistical tests not related to RNA sequencing, the Kruskal-Wallis test was used to compare multiple groups. To account for multiple comparisons, only if the Kruskal-Wallis test was significant would a Wilcoxon rank-sum test (Mann-Whitney U) or Fisher’s exact test be used for two-group comparisons. A two-way analysis of variance (ANOVA) was used for comparisons between groups with repeated measures over time. The threshold for statistical significance was set at <0.05. Analyses were conducted using GraphPad Prism 8 (San Diego, CA, USA) and Stata 15 (College Station, TX, USA).

## Results

### Donor demographics and functional assessment

Eleven discarded DCD livers were included in the control cohort and stratified using predefined criteria for demonstrating adequate hepatocellular function. **Table 1** displays donor and liver characteristics between 6 livers demonstrating adequate hepatocellular function (AHF) and five livers with inadequate hepatocellular function (IHF). No significant differences were found between groups, though there was a trend toward longer cold ischemic time in the AHF group and higher donor body mass index in the IHF group. Median DRI was 2.4 [2.2,2.4] in IHF livers compared to 2.05 [1.9,2.2] in AHF livers (p=0.14). Donor demographics for the five emricasan-treated livers are also shown. Livers EM1 and EM2 were from young DCD donors with relatively low DRIs turned down due to logistic reasons and inability to find a suitable recipient. Livers EM3-5 were from older donors with extended warm and cold ischemic times, and therefore had notably high DRIs.

**Table 1:** Discarded liver donor demographics.

	EmricasanN=5					AHFN=6	IHFN=5	AHF *vs*. IHF P-value
**Liver #**	EM1	EM2	EM3	EM4	EM5			
**DCD?**	Y	Y	Y	Y	Y	6 (100%)	5 (100%)	
**tWIT**	22	23	32	23	53	27.5 (24–28)	24 (23-34)	0.93
**fWIT**	9	8	7	12	13	8.5 (8-10)	11 (9-11)	0.36
**CIT**	347	553	758	703	679	625.5 (458-697)	358 (357-367)	0.082
**Age**	28	26	60	50	59	58.5 (40-60)	56 (55-59)	0.85
**Gender**	M	M	F	F	F	5M, 1F	2M, 3F	0.24
**BMI**	38.7	19.8	31.2	28.3	31.9	26 (24.5-28.3)	32.9 (29.9-40.2)	0.068
**Weight (kg)**	129.5	61.7	90.5	74.9	84.8	80.6 (77.6-87)	90.9 (74.1-100)	0.86
**Macrosteatosis**	30	0	2	0	0	3.5 (0-5)	35 (0-40)	0.35
**Microsteatosis**	10	5	0	30	0	3.8 (0-50)	5 (0-55)	0.85
**AST**	32	38	134	78	27	41.5 (39-57)	98.5 (41.5-366)	0.39
**ALT**	22	27	40	47	32	32.5 (22-58)	100.5 (25.5-317.5)	0.52
**Total bilirubin**	1.2	0.8	0.6	0.2	0.2	0.75 (0.4-1)	0.35 (0.25-0.45)	0.086
**ALP**	72	48	76	104	83	58 (36-95)	99 (94-494)	0.12
**DRI**	1.74	1.47	3.56	3.04	2.83	2.05 (1.9-2.2)	2.4 (2.2-2.4)	0.14

Median with interquartile range shown for continuous data. Wilcoxon rank-sum test or Fisher’s exact test used for group comparisons. AHF, adequate hepatocellular function group; IHF, inadequate hepatocellular function group; DCD, donation after circulatory death; tWIT, total warm ischemic time (extubation to cold flush) in minutes; fWIT, functional warm ischemic time (asystole to cold flush) in minutes; CIT, cold ischemia time in minutes; BMI, body mass index (kg/m^2^); steatosis expressed in percentages; DRI, donor risk index. Terminal values prior to procurement shown for AST (aspartate aminotransferase), ALT (alanine aminotransferase), ALP (alkaline phosphatase).

**Figure 2:**
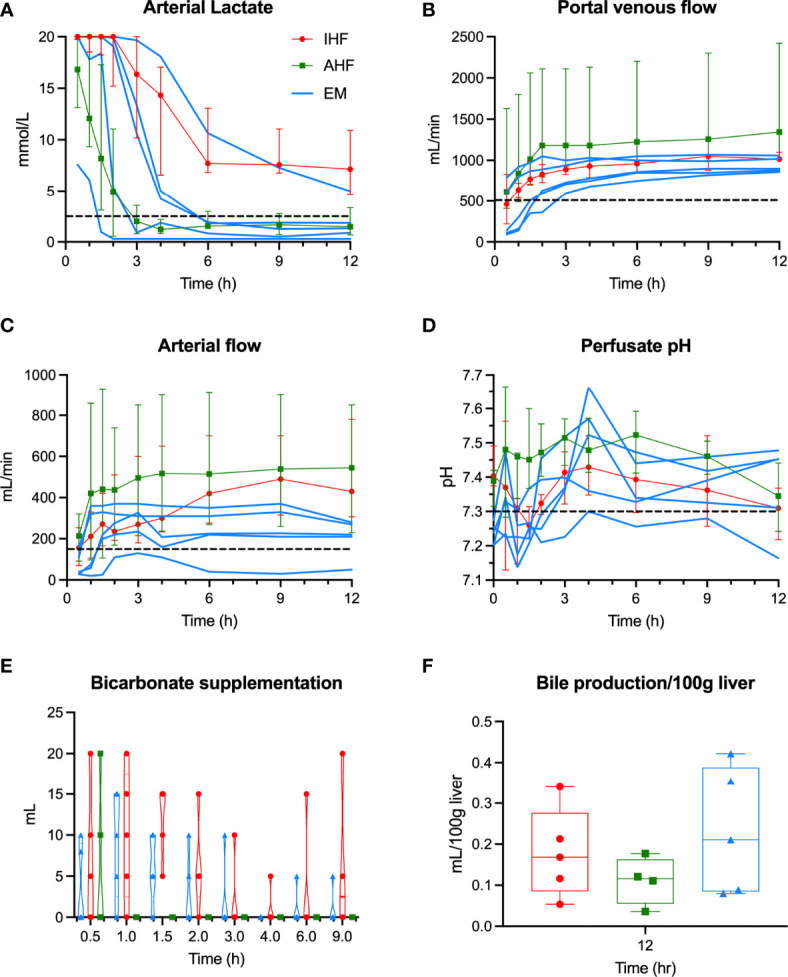
Hepatocellular perfusion metrics during NMP. Individual liver data for emricasan-treated livers (EM) shown in blue. Median and interquartile ranges shown for AHF (green) and IHF (red) groups. Dashed black line shows the cutoff used to determine adequate hepatocellular function for **(A)** arterial lactate, **(B)** portal flow, **(C)** arterial flow, and **(D)** perfusate pH. **(E)** Volume of bicarbonate supplementation required to maintain pH>7.3 show. Only the inadequate functioning EM liver continued to require bicarbonate after 2 hours of NMP. **(F)** Bile production shown for each group. No significant difference was seen though to livers in the AHF group were omitted due to technical issues with bile collection. NMP, normothermic machine perfusion; AHF, adequate hepatocellular function; IHF, inadequate hepatocellular function; EM, emricasan.

With respect to hepatocellular function (**Figure 2**), four emricasan-treated livers demonstrated adequate hepatocellular function, including EM3 and EM4 (DRI 3.56 and 3.04, respectively). Low DRI livers EM1 and EM2 rapidly cleared lactate in similar fashion to AHF livers. Livers EM3 and EM4 initially displayed delayed lactate clearance within the first two hours of NMP similar to IHF livers, but subsequently were able to demonstrate adequate lactate clearance by 6 hours of NMP. Liver EM5, with the longest WIT, demonstrated IHF and failed to meet the lactate threshold despite a steady decrease in perfusate lactate throughout NMP (**Figure 2A**). Like AHF livers, EM1-4 livers demonstrated stable vascular flows and perfusate pH (**Figures 2B–D**). Only liver EM5 required bicarbonate supplementation after 2 hours of NMP to maintain pH above 7.3 (**Figure 2E**). Bile production was similar between the three groups (**Figure 2F**).

Analysis of cholangiocellular metrics demonstrated relatively adequate function in EM1 and EM2, which had the shortest ischemic times compared to EM3-5. Comparison of EM livers to the control groups was limited by differences in collection times and lack of corresponding perfusate chemistries (**Figure S1**).

### Principal component analysis (PCA) and gene expression

**Figure 3:**
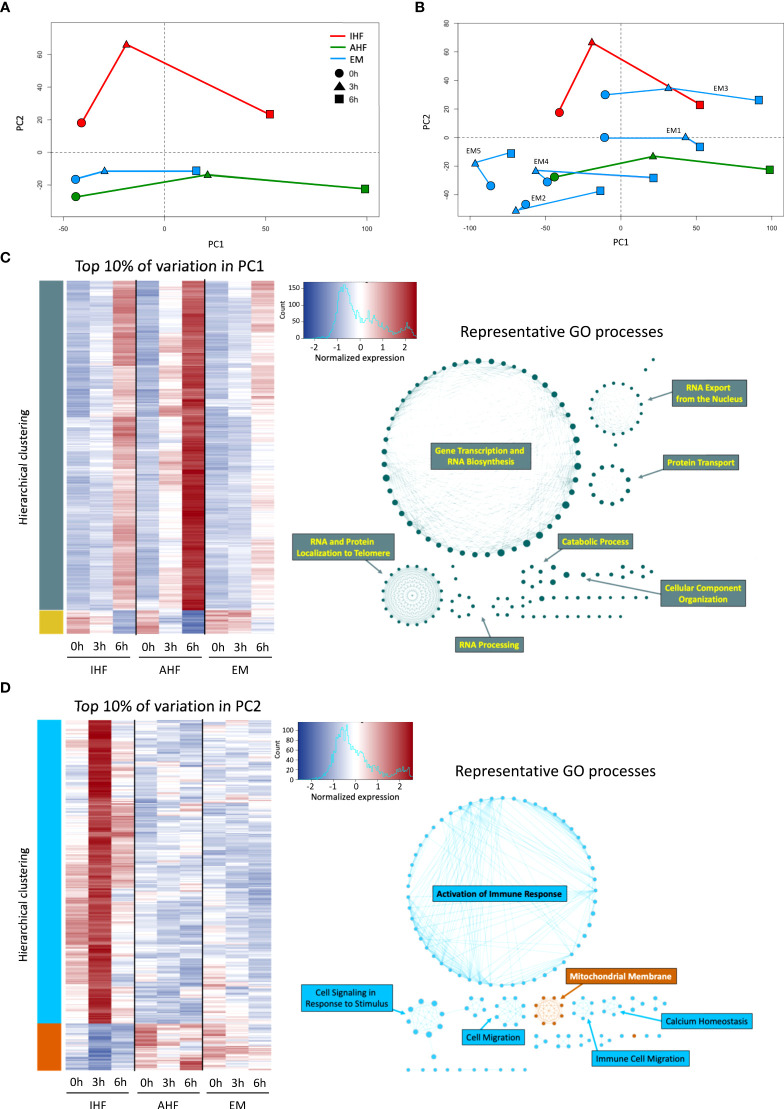
Principal component and gene ontology analysis of transcriptional changes during NMP. PCA of EM, AHF, and IHF groups for PC1 and PC2 at 0 (pre-perfusion), 3, 6 hours of NMP shown in **(A)** composite form and **(B)** with individual EM livers. PC2 clearly distinguishes AHF from IHF livers, whereas PC1 captures time-dependent transcriptional changes. The worst functioning liver (EM5) demonstrates the least transcriptional change during NMP. **(C)** Hierarchical clustering heatmap of the top 10% of genes accounting for the variation in PC1. GO network analysis indicates PC1 is enriched for gene expression accounting for DNA transcription and RNA translation (teal networks), which is upregulated in all three groups during NMP. D) The top 10% of genes accounting for the variation in PC2 show remarkable differences between the three groups. GO network analysis indicates significant upregulation of genes involved in immune response activation and cell migration (light blue networks) in the IHF group compared to AHF and EM groups. AHF and EM groups demonstrated comparatively increased expression of genes related to mitochondrial membrane integrity (orange network) compared to IHF livers. AHF, adequate hepatocellular function; IHF, inadequate hepatocellular function; EM, emricasan; PC, principal component.

PCA demonstrated notable transcriptional trends among the 3 groups (**Figures 3A, B**). Principal component 1 (PC1) captured the time-dependent changes in gene expression. Livers with AHF demonstrated consistent large transcriptional shifts at 3 and 6 hours of perfusion while these changes were dampened in the IHF and emricasan-treated livers especially at the 3h timepoint. Analysis of the top 10% of genes accounting for the variation in PC1 revealed Gene Ontology (GO) processes related to RNA transcription, RNA processing, and protein translation. Compared to AHF and IHF livers, EM livers demonstrated muted upregulation of genes within the PC1 subset (**Figure 3C**). On the other hand, PC2 appeared to distinguish AHF from IHF livers. EM livers clustered closer to AHF livers on the PC2 axis. Analysis of the top 10% of genes accounting for PC2 revealed marked differences between groups both prior to initiation of and during NMP. This gene subset demonstrated functional enrichment of GO processes related to immune response activation, immune cell migration, and the mitochondrial membrane. The gene expression heatmap demonstrates strong upregulation of genes categorized under the immune response phenotype in the IHF group after initiation of perfusion. In contrast, AHF and EM livers demonstrate more robust activation of genes associated with the mitochondrial membrane phenotype and minimal immune response (**Figure 3D**). Transcriptional changes accounting for PC3 were enriched for GO processes related to mitosis and microtubule organization (**Figure S2**). Analysis of the canonical pathways and upstream regulators obtained from Ingenuity Pathway Analysis (IPA) are shown in **Figure S3**.

**Figure 4:**
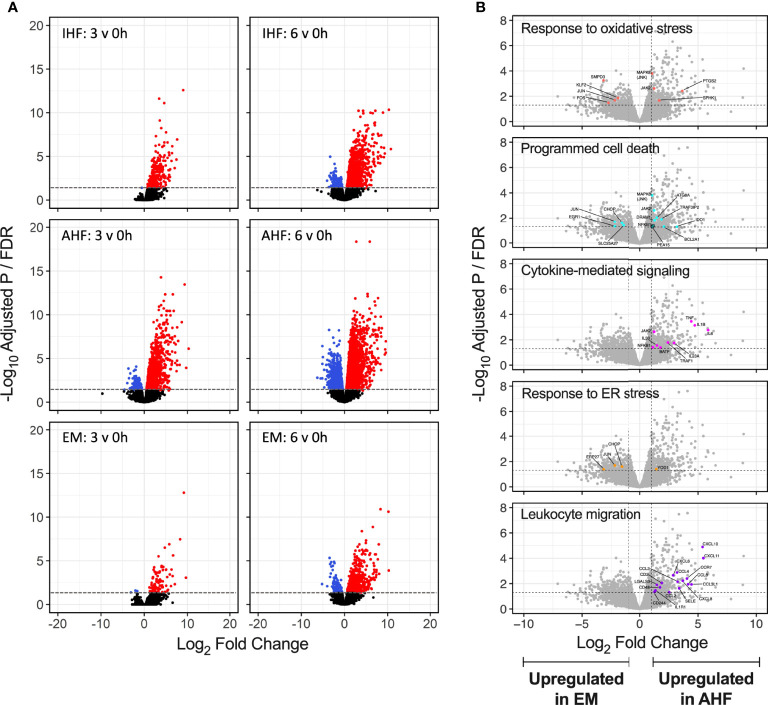
Volcano plots of differentially expressed genes during NMP. **(A)** Significant upregulated (red) and downregulated (blue) genes shown for each group at 3 and 6 hours of NMP compared to pre-perfusion. The absolute number of differentially expressed genes appeared lower in the EM compared to AHF and IHF groups. **(B)** Select GO term volcano plots shown for comparison of EM to AHF groups at 6 hours of NMP. Significant differentially expressed genes within each GO term are shown in color and labelled. Genes related to inflammatory signaling and leukocyte migration were significantly upregulated in AHF compared to EM livers. Horizontal dashed line indicates the adjusted P value (false discovery rate) cutoff of 0.05. Vertical dashed lines indicate the log_2_ fold change cutoff -1 and 1. NMP, normothermic machine perfusion; GO, gene ontology; AHF, adequate hepatocellular function; IHF, inadequate hepatocellular function; EM, emricasan; ER, endoplasmic reticulum.

Volcano plots of differentially expressed genes after 3 and 6 hours of NMP compared to pre-perfusion biopsies demonstrated robust up- and down-regulation of genes in the AHF livers. In comparison, IHF livers demonstrated significant expression of a smaller number of genes. Emricasan-treated livers displayed a markedly smaller set of DEGs compared to both control groups (**Figure 4A**). To further detail differences between AHF and EM livers, volcano plots comparing gene expression after 6 hours of NMP between the two groups were annotated for select GO processes. Compared to EM livers, the AHF group demonstrated more robust activation of genes associated with oxidative stress, cytokine-mediated signaling, programmed cell death, and leukocyte migration (**Figures 4BF**). Similar patterns were seen when comparing EM and IHF livers. No significant differences were noted between AHF and IHF groups given equally robust expression of implicated genes within the GO processes of interest (**Figure S4**)

### Adjunct delivery of caspase inhibitor mitigates IRI during liver NMP

**Figure 5:**
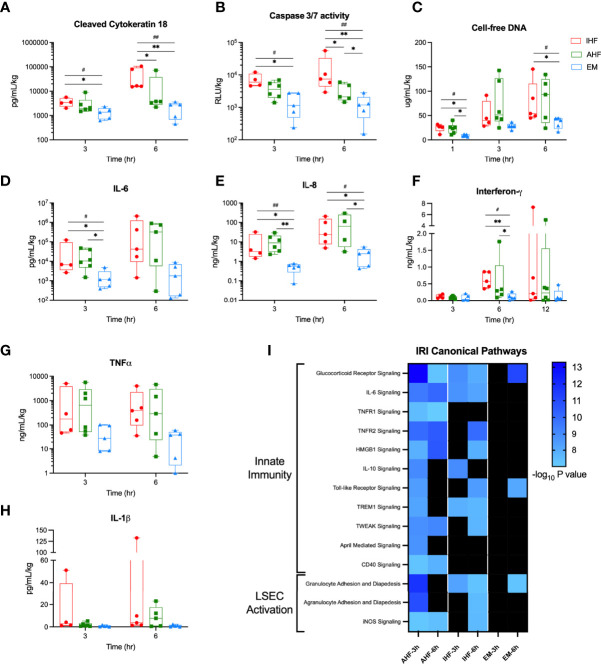
Perfusate cell death and cytokine analysis. Addition of emricasan to NMP resulted in significantly lower perfusate levels of the apoptosis biomarkers **(A)** cleaved cytokeratin 18 and **(B)** caspase-3/7 enzyme activity. **(C)** Cell-free DNA, a DAMP, was significantly lower in the perfusate of EM livers at 1 and 6 hours. Levels of pro-inflammatory cytokines **(D)** interleukin-6 (IL-6), **(E)** interleukin-8 (IL-8), and **(F)** interferon-*γ* were also significantly lower in EM livers compared to control groups. Perfusate levels of **(G)** tumor necrosis factor-*α* (TNF-*α*) and **(G)** interleukin-1*β* (IL-1 *β*) were qualitatively lower but did not reach significance. I) Heatmap of canonical pathways involved in ischemic-reperfusion injury (IRI). The significance threshold was set to -log10 P value > 7 for comparison of 3- and 6-hour gene sets to the pre-perfusion (0 hour) baseline for each group. Kruskal-Wallis test: ## P<0.01, # P<0.05; Wilcoxon ranksum test: ** P<0.01, * P<0.05. NMP, normothermic machine perfusion; AHF, adequate hepatocellular function; IHF, inadequate hepatocellular function; EM, emricasan.

Analysis of perfusate plasma samples for apoptosis biomarkers showed significantly higher circulating levels of cleaved cytokeratin 18 and caspase-3/7 activity after 6 hours of NMP in IHF compared to AHF livers. Addition of emricasan significantly decreased evidence of apoptosis after both 3 and 6 hours of NMP. Circulating cell-free DNA, a damage-associated molecular pattern (DAMP), was also significantly reduced in the EM livers compared to control groups (**Figures 5A–C**).

To determine the consequences of emricasan treatment on extracellular signaling proteins, plasma levels of numerous pro-inflammatory cytokines were evaluated. EM livers demonstrated significantly lower levels of interleukin-6 (IL-6), interleukin-8 (IL-8), and interferon-γ (IFNG) compared to AHF and IHF groups (**Figures 5DF**). Circulating tumor necrosis factor-α (TNFα) and interleukin-1β (IL-1β) were qualitatively lower but failed to meet statistical significance (**Figures 5G, H**). Histopathologic analysis demonstrated a significant time-dependent increase in inflammatory cell infiltrate and necrosis among the three groups but no difference between groups. Qualitatively, EM livers did not demonstrate an increase in inflammatory cells over time compared to IHF livers (**Figure S5**).

Given the decreased pro-inflammatory cytokine profile in the perfusate of EM livers, Ingenuity Pathway Analysis was used to evaluate the downstream signaling effect of these findings. EM livers demonstrated minimal enrichment of IRI canonical pathways associated with innate immunity and sinusoidal endothelial cell activation (**Figure 5I**). IHF livers also demonstrated a pattern of delayed activation of these canonical pathways at 6 hours of NMP whereas AHF livers appeared to show early activation at 3 hours. Moreover, downstream gene target expression analysis of the above pro-inflammatory upstream regulators revealed categorically fewer differentially expressed genes in EM livers compared to AHF and IHF groups (**Figure S6**).

### Stress response mechanisms in the setting of caspase inhibition

**Figure 6:**
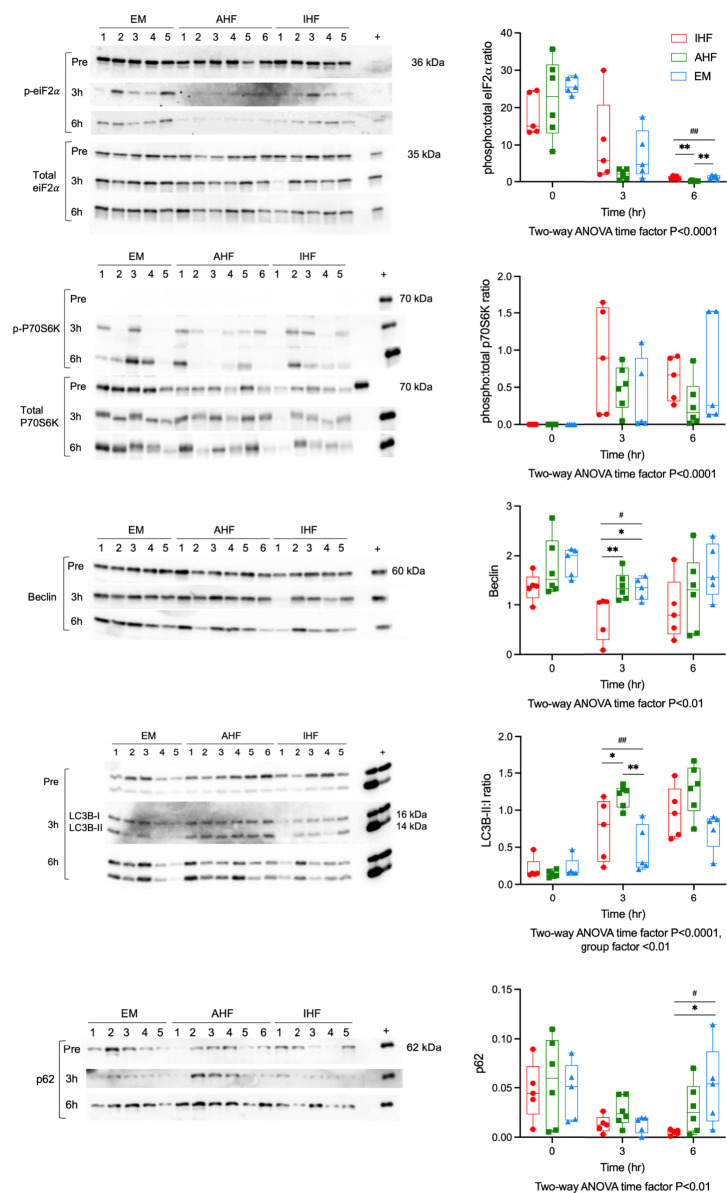
Autophagy and the unfolded protein response during liver NMP. Western immunoblots of **(A)** phosphorylated to total protein ratio of eiF2*α*, **(B)** phosphorylated to total protein ratio P70S6K, **(C)** Beclin, **(D)** LC3B-II to LC3B-I ratio, and **(E)** p62. Kruskal-Wallis test: ## P<0.01, # P<0.05; Wilcoxon ranksum test: ** P<0.01, * P<0.05. NMP, Normothermic machine perfusion; AHF, adequate hepatocellular function; IHF, inadequate hepatocellular function; EM, emricasan; eiF2*α*, eukaryotic initiation factor 2*α*; P70S6K, P70 S6 kinase; LC3B, microtubule-associated protein 1 light chain 3 beta.

We have previously shown that functional livers demonstrate a more robust homeostatic stress response including activation of autophagy compared to inadequately functioning livers. However, when the cellular stress response is overwhelmed, crosstalk between autophagy and apoptosis initiates programmed cell death (5). We hypothesized that caspase inhibition would bolster cell survival and upregulate autophagy, thereby improving hepatocellular function. Therefore, we performed Western immunoblotting for key proteins involved in regulation of these processes. Eukaryotic initiation factor 2α (eIF2α) is phosphorylated in response to misfolded proteins accumulating in the endoplasmic reticulum (ER). This phosphorylation of eIF2α leads to initiation of antioxidant signaling, repression of global protein translation and induction of autophagy. Western immunoblotting revealed high levels of phospho-eIF2α pre-perfusion in all groups. After initiation of perfusion all groups demonstrated a significant time-dependent decrease in phosphorylation. After 6 hours of perfusion, AHF livers had lower phospho:total-eiF2α ratios (0.21, IQR 0.14-0.31) compared to IHF (0.78, IQR 0.69-1.58, P<0.01 *vs*. AHF) and EM livers (1.22, IQR 0.73-.78, P<0.01 *vs*. AHF) (**Figure 6A**). The mechanistic Target of Rapamycin (mTOR) pathway also plays a key role in regulation of autophagy with activation of mTORC1 leading to suppression of autophagy. Phosphorylation of ribosomal protein S6 kinase (P70S6K), a downstream target of mTORC1, was not detectable pre-perfusion. NMP resulted in a significant time-dependent increase in phospho:total-P70S6K ratios in all groups (**Figure 6B**). There was a trend toward higher levels in the IHF compared to AHF and emricasan treated livers. However, this did not reach significance due to the variation in individual livers. Proteins directly involved in autophagic flux were investigated next. Beclin, a marker of autophagy induction, demonstrated a time-dependent decrease during perfusion. Beclin protein content in IHF livers after 3 hours of NMP was significantly lower compared to AHF and EM groups (**Figure 6C**). This difference was qualitatively apparent at 6hr although it was no longer statistically significant. Following initiation of autophagy, microtubule-associated protein 1 light chain 3 beta (LC3B) is converted from its inactive form LC3B-I to LC3B-II through lipidation by the autophagy machinery. LC3B-II then associates with the lipid membranes of the phagophore to form the autophagosome. LC3B-II:I ratios were significantly higher in AHF livers (1.33, IQR 0.99-1.57) at 3hr compared to IHF (0.96, IQR 0.64-1.29, P<0.05 *vs*. AHF) and emricasan livers (0.85, IQR 0.51-0.89, P<0.01 *vs*. AHF). The ratio increased in the IHF livers at 6hr such that there was no difference between IHF and AHF livers (**Figure 6D**). We confirmed the LC3B results using immunohistochemistry (**Figure S7**). Pre-perfusion biopsies demonstrated pan-cytosolic staining in all groups. In AHF livers, LC3B was localized to a granule staining pattern at 3 and 6hr indicative of LCB-II association with the autophagosome. This shift in staining pattern was not observed in the IHF group and variable in emricasan livers. p62 content was investigated to determine whether autophagy was potently inhibited in IHF or EM livers (**Figure 6E**). A trend toward lower levels of p62 was observed in all groups at 3hr compared to pre-perfusion. However, there was a notable divergence in p62 expression at 6hr of perfusion with IHF livers maintaining very low expression compared to EM livers.

Finally, we sought to determine if pan-caspase inhibition resulted in off-target activation of other cell death pathways such as necroptosis (6, 19). To assess whether treatment with emricasan resulted in compensatory activation of necroptosis, we probed for phosphorylation of mixed lineage kinase domain-like (MLKL). MLKL is the terminal kinase in the necroptotic cell death program. Phosphorylation of MLKL is believed to lead to its oligomerization, transport to the membrane, and membrane destruction. Phospho-MLKL was below the limit of detection in all groups before and during perfusion, suggesting that treatment with emricasan did not lead to induction of necroptosis (**Figure S8**).

## Discussion

Pathologic apoptosis, represented by an imbalance between regulated cell death and cell renewal, is an unregulated process that can be sustained and injurious (7). Release of intra-cellular contents, including DAMPs and cytokines, activate immune cells and propagate the cycle of cell death and inflammation. In this study of discarded livers subjected to NMP, livers with IHF were characterized by abundant apoptotic cell death, robust and sustained activation of innate immunity, and decreased activation of ER stress response pathways (autophagy) when compared to livers with AHF. Addition of an irreversible pan-caspase inhibitor, emricasan, to the perfusate composition resulted in significantly lower levels of apoptotic cell death and mitigation of innate immune responses during NMP. Comprehensive analysis of transcriptomic data indicate addition of emricasan resulted in suppression of gene expression related to innate immunity such as cytokine-mediated signaling and leukocyte migration, as well as decreased concentrations of DAMPs and pro-inflammatory cytokines in the circulating perfusate. Transcriptional changes were seen at the level of both upstream regulators and downstream target gene expression.

Early applications of emricasan in rodent LT models demonstrated improved survival when drug was given at the time of liver procurement (10–12). In a subsequent randomized clinical trial, Baskin-Bey et al. demonstrated significantly lower AST and ALT levels in LT recipients when emricasan was added to the preservation flush solution compared to a placebo group, though the trial was not powered to detect differences in graft survival or EAD (13). Despite these promising results, routine clinical application of emricasan never reached wider use likely owing to the complexity of incorporating drug therapies into the clinical workflow at the time of organ procurement. The advent of perfusion technologies as a platform for organ rehabilitation is likely to provide a solution to these obstacles as organs can be dynamically preserved, monitored, and treated *ex situ* prior to implant (20). This study is a step forward in this direction, demonstrating that treatment of discarded DCD livers with emricasan during NMP after a period of cold and warm ischemia mitigated injury driven by innate immune and pro-inflammatory responses during *ex situ* preservation.

The physiologic benefits of emricasan-adjunct NMP are likely multifactorial. Studies of individual caspase enzymes in the last decade have revealed varied functionality, with some acting as initiators of cell death (caspase 8,9,10) and others as executioners involved in end-stage proteolysis (caspase 3,6,7). A third class named inflammatory caspases (caspase 1,4,5) play a significant role in inflammasome activation and IL-1*β* release, often characterized as pyroptosis (6, 19). Inhibition of inflammatory caspases by emricasan may be one reason EM-treated livers had uniformly depressed IL-1*β* perfusate levels and markedly suppressed transcriptional enrichment of innate immune responses. Additional studies are needed to determine whether equivalent results can be obtained with specific inflammatory caspase inhibitors versus the pan-caspase inhibitor used here. A narrower therapeutic target may also minimize off-target consequences. For these reasons, we cannot definitively conclude that inhibition of apoptosis in of itself was the mechanism by which *ex situ* innate immune responses were mitigated, or whether inhibition of pyroptosis had a significant beneficial effect. However, necroptosis does not appear to have a major role during liver NMP, nor does it appear to be unintentionally activated by pan-caspase inhibition.

Despite these limitations, prevention of unregulated apoptosis (and pyroptosis) and thus release of DAMPs by injured hepatocytes likely minimizes activation of resident immune cells and further cytokine release. This appears to disrupt the vicious cycle wherein injured hepatocytes undergo unregulated apoptosis and activate a cascade of cytokine-driven innate immune responses that in turn drive further cell death. Using a rat liver NMP model, Scheuermann et al. likewise demonstrated higher cytokine and DAMP concentrations with resultant downstream signaling consequences, including apoptosis. Less inflammatory signaling was seen when rat livers were perfused at lower temperatures (21). Furthermore, it is of interest that the cytokine signature seen in IHF and some AHF livers was redolent of the pattern seen in sera of liver transplant recipients who experienced IRI in a study by Sosa et al. (18). In contrast, the suppressed proinflammatory cytokine signature seen in EM livers more closely resembled non-IRI livers. However, histopathology did not demonstrate a significant difference in inflammatory cell infiltrate, necrosis, or reperfusion injury between individual groups. This may be a limitation of sample size or the scoring system, which was extrapolated from post-LT reperfusion biopsies, though it warrants more cautious interpretation of these results.

We further investigated ER stress responses to delineate potential crosstalk between apoptosis and autophagy. Prior studies have shown when cellular stress overwhelms autophagy, cells are directed toward apoptosis (5). We therefore hypothesized that inhibiting apoptosis would increase flux through autophagy in ischemic-injured hepatocytes, thereby promoting cell recovery and improving hepatocellular function. In fact, we found a dampened autophagic response. AHF livers demonstrated significantly more activation of autophagy, corroborating our findings from a recent smaller study (3). On the other hand, EM livers demonstrated comparatively moderate autophagic flux, with an appropriate decrease in the ER stress marker phospho-eiF2*α*, a modest increase in LC3B-II:I ratios, and a cyclic reactivation of autophagy inhibition by 6 hours of NMP. It is possible that the dampened inflammatory response due to emricasan treatment does not require such a strong cellular stress response. A more likely consideration is that caspase inhibition was too far downstream and did not achieve the intended effect on autophagy. Targeting proteins more upstream at the nexus between autophagy and apoptosis may be a better therapeutic option (22).

With respect to rehabilitation of liver hepatocellular function, it is not possible to draw definitive conclusions given the variability between EM livers. Isolated examination of EM-3 and EM-4 livers (and to a lesser extent EM-5) appear to demonstrate better than expected lactate clearance, based on the initial slow clearance followed by a more rapid drop. We were unable to evaluate post-transplant function of the research livers as no perfusion device had received regulatory approval in the United States at the time of this study. Alternatively, whole blood reperfusion studies could have been incorporated into the emricasan-treated livers but would have been limited by our use of retrospectively reanalyzed samples for the control groups. However, the feasibility of a clinical trial is no longer a barrier with recent FDA approval of two NMP devices.

Another limitation worth discussion is the wide variability seen in human liver studies. The relatively small group size in this study limited our ability to make more definitive conclusions. Despite this, we were able to demonstrate statistically significant differences in both transcriptional and protein analyses with clinically important implications for real-world practice. Future studies with a larger study population may be better suited for anticipated clinical trials given the resource-intensive nature of preclinical discarded human research. Another consideration was the apparent non-superiority in improving cholangiocellular function, as emricasan did not appear to improve IRI in cholangiocytes. This is a known limitation of end-ischemic NMP in DCD livers (23) and a precursor period of hypothermic oxygenated perfusion with or without controlled rewarming is likely required to prevent detrimental IRI in cholangiocytes (2, 24). The observed trend toward higher bile production in emricasan livers likely reflects the preserved hepatocellular function of bile secretion but not the cholangiocellular function of bile detoxification and alkalinization (25). In contrast, one factor that favors the wider application of emricasan is its excellent safety and tolerance profile, having been demonstrated in clinical trials for both liver transplant and non-alcoholic steatohepatitis (13, 26). Finally, the endpoints (cleaved cytokeratin 18, caspase-3/7 activity) used to determine therapeutic efficacy (26, 27) demonstrated a significant measurable difference within a clinically relevant perfusion timeframe, making incorporation of adjunct therapy a viable option during routine NMP. For these reasons, translation of these preclinical findings into a pilot clinical trial appears feasible. An initial series of standard criteria donor livers undergoing emricasan-adjunct NMP followed by LT would allow drug safety and adverse event monitoring. This could be followed by a larger randomized clinical trial with attention paid to early allograft dysfunction and graft survival as endpoints. In conclusion, therapeutic pan-caspase inhibition during liver NMP mitigates innate immune and pro-inflammatory responses in the *ex situ* setting and may be a valuable tool for improving hepatocellular function prior to transplantation.

## Data availability statement

The datasets presented in this study can be found in online repositories. The names of the repository/repositories and accession number(s) can be found in the article/**Supplementary Material**.

## Ethics statement

The studies involving human participants were reviewed and approved by Massachusetts General Hospital Institutional Review Board and Lifespan Institutional Review Board. The patients/participants provided their written informed consent to participate in this study.

## Author contributions

SR: conceptualization, investigation, methodology, formal analysis, funding acquisition, visualization, writing – original draft, and writing – review and editing. JSant: investigation, data curation, software, methodology, formal analysis, and visualization. AO: investigation, data curation, software, methodology, formal analysis, and visualization. MH: investigation, methodology, formal analysis, and visualization. SB: investigation and formal analysis.

TC: investigation and formal analysis. RV: investigation and formal analysis. IR: investigation, methodology, and formal analysis. AS: investigation, methodology, and formal analysis. JM: resources, supervision, funding acquisition, and writing – review and editing. PG: resources, supervision, funding acquisition, and writing – review and editing. KU: resources, supervision, funding acquisition, project administration, and writing – review and editing. JSand: resources, methodology, formal analysis, funding acquisition, project administration, supervision, and writing – review and editing. HY: resources, methodology, formal analysis, funding acquisition, project administration, supervision, and writing – review and editing. All authors contributed to the article and approved the submitted version.

## Funding

AO and JSand are supported by National Institute of Environmental Health Sciences (T32ES007272). SR, HY, and RV are supported by the Massachusetts General Hospital Executive Committee on Research. This research was funded by the National Institutes of Diabetes and Digestive and Kidney Diseases (R01DK096075, R01DK107875, R01DK114506), the National Science Foundation (EEC 1941543, ATP-Bio), and the Rhode Island Hospital/Brown University Department of Pediatrics.

## Acknowledgments

We would like to thank the donors, their families, and the organ procurement organization staff for making this work possible.

## Conflict of interest

SR, HY, and KU are inventors on pending patents relevant to this study and have a provisional patent application relevant to this study. KU has a financial interest in Sylvatica, a company focused on developing organ preservation technology. KU’s interests are managed by the Massachusetts General Hospital and Mass General Brigham in accordance with their conflict of interest policies.

The remaining authors declare that the research was conducted in the absence of any commercial or financial relationships that could be construed as a potential conflict of interest.

## Publisher’s note

All claims expressed in this article are solely those of the authors and do not necessarily represent those of their affiliated organizations, or those of the publisher, the editors and the reviewers. Any product that may be evaluated in this article, or claim that may be made by its manufacturer, is not guaranteed or endorsed by the publisher.

## Supplementary material

Supplementary Figure 1 Cholangiocellular functional criteria during liver NMP. Various cholangiocellular functional criteria demonstrated. Technical issues with bile collection prevented accurate measurement of bile in two livers from the AHF group. Bile pH and bicarbonate is shown collectively for all available livers. Additional functional data comparing bile and perfusate glucose, pH, and bicarbonate is shown only for EM livers as equivalent data points were not available for the control livers. NMP, normothermic machine perfusion; AHF, adequate hepatocellular function; IHF, inadequate hepatocellular function; EM, emricasan.

Supplementary Figure 2 Gene expression and ontology analysis of principal component 3 (PC3). **(A)** Combined and **(B)** individual principal component plots for PC3 across PC1. **(C)** Hierarchical clustering heatmap of the top 10% of genes accounting for the variation in PC3. GO network analysis indicates PC3 is enriched for gene expression accounting for mitosis (forest green networks) and DNA transcription (beige networks). In contrast to AHF and IHF livers, EM livers appear to demonstrate overall upregulation of mitosis-related genes. AHF, adequate hepatocellular function; IHF, inadequate hepatocellular function; EM, emricasan.

Supplementary Figure 3 Ingenuity Pathway Analysis (IPA) of differentially expressed genes. Dot plots contain significant **(A)** canonical pathways (CP) and **(B)** upstream regulators (UR) for the arbitrary P value cut-off indicated in the legend (10^-7^ for CPs and 10^-14^ for URs). Results are shown for differentially expressed genes at 3 and 6 hours of NMP compared to the pre-perfusion (0 hour) baseline. The size of each dot represents the number of downstream target genes in our gene set that were in the IPA gene set for that CP or UR. The color of each dot represents the P value for the CP or UR. NMP, normothermic machine perfusion; AHF, adequate hepatocellular function; IHF, inadequate hepatocellular function; EM, emricasan.

Supplementary Figure 4 Selective volcano plots for all group comparisons. Individual time-point comparisons at 3 and 6 hours of NMP among all groups are demonstrated for the select gene ontology processes demonstrated in . Differentially expressed genes meeting the false discovery rate (FDR) > 0.05 threshold are shown in color and labeled. NMP, normothermic machine perfusion; AHF, adequate hepatocellular function; IHF, inadequate hepatocellular function; EM, emricasan.

Supplementary Figure 5 Histopathologic reperfusion injury during NMP. Blinded pathologists scored 0, 3, and 6 hours biopsy samples for **(A)** inflammatory cell infiltrate, **(B)** necrosis, and **(C)** reperfusion injury (a composite of **A** and **B**). The two-way ANOVA time factor was statistically significant for all three evaluated histologic markers. No significant differences were seen between groups. NMP, normothermic machine perfusion; AHF, adequate hepatocellular function; IHF, inadequate hepatocellular function; EM, emricasan; ANOVA, analysis of variance.

Supplementary Figure 6 Downstream target gene expression for selected proinflammatory cytokine. Proinflammatory cytokines from were analyzed as upstream regulators in IPA for downstream target gene expression. EM livers demonstrate categorically lower enrichment of target genes compared to IHF and AHF groups. NMP, normothermic machine perfusion; AHF, adequate hepatocellular function; IHF, inadequate hepatocellular function; EM, emricasan; IPA, Ingenuity Pathway Analysis; TNF, tumor necrosis factor; IL1B, interleukin-1 *β*; IFNG, interferon- *γ*; CXCL8, C-X-C motif chemokine ligand 8 (also known as interleukin-8).

Supplementary Figure 7 LC3B Immunohistochemistry. Immunohistochemistry for LC3B demonstrating patterns of autophagosome assembly during perfusion among the three groups. AHF livers demonstrate active autophagy at 3 and 6 hours of NMP as indicated by the transition from pancytosolic to granular LC3B staining pattern. In contrast, IHF livers demonstrated more sporadic granular LC3B staining after initiation of NMP. EM livers had variable staining with some livers demonstrating granular LC3B staining after 3 hours with return to pancytosolic staining by 6 hours of NMP. 100 micron scale bar shown. NMP, Normothermic machine perfusion; AHF, adequate hepatocellular function; IHF, inadequate hepatocellular function; EM, emricasan; LC3B, microtubule-associated protein 1 light chain 3 beta.

Supplementary Figure 8 Necroptosis and protein controls during NMP. **(A)** Western immunoblot analysis of phosphorylated and total levels of the necroptosis marker mixed lineage kinase domain-like (MLKL). Phosphorylated MLKL protein was below the level of detection at pre-perfusion and after 6 hours of NMP. **(B)** *β*-actin and glyceraldehyde-3-phosphate dehydrogenase (GAPDH) levels for all three groups. NMP, normothermic machine perfusion; AHF, adequate hepatocellular function; IHF, inadequate hepatocellular function; EM, emricasan.

Supplemental Methods Detailed description of machine perfusion techniques, RNA sequencing, bioinformatics analysis, protein, and immunohistochemistry studies performed.

Supplementary Data Sheet 1 Full list of gene ontology (GO) processes demonstrating enrichment used to create the network diagrams for principal component 1-3 in and **Figure S2**.

## References

[1] Nasralla D Coussios CC Mergental H Akhtar MZ Butler AJ Ceresa CDL. A randomized trial of normothermic preservation in liver transplantation. Nature (2018) 557(7703):50–6. doi: 10.1038/s41586-018-0047-9 29670285

[2] van Rijn R Schurink IJ de Vries Y van den Berg AP Cortes Cerisuelo M Darwish Murad S. Hypothermic machine perfusion in liver transplantation - a randomized trial. N Engl J Med (2021) 384(15):1391–401. doi: 10.1056/NEJMoa2031532 33626248

[3] Ohman A Raigani S Santiago JC Heaney MG Boylan JM Parry N. Activation of autophagy during normothermic machine perfusion of discarded livers is associated with improved hepatocellular function. Am J Physiol Gastrointest Liver Physiol (2022) 322(1):G21–33. doi: 10.1152/ajpgi.00266.2021 PMC8698515 34730028

[4] Brenner C Galluzzi L Kepp O Kroemer G. Decoding cell death signals in liver inflammation. J Hepatol (2013) 59(3):583–94. doi: 10.1016/j.jhep.2013.03.033 23567086

[5] Fairlie WD Tran S Lee EF. Crosstalk between apoptosis and autophagy signaling pathways. Int Rev Cell Mol Biol (2020) 352:115–58. doi: 10.1016/bs.ircmb.2020.01.003 32334814

[6] Galluzzi L Lopez-Soto A Kumar S Kroemer G. Caspases connect cell-death signaling to organismal homeostasis. Immunity (2016) 44(2):221–31. doi: 10.1016/j.immuni.2016.01.020 26885855

[7] Guicciardi ME Gores GJ. Apoptosis: a mechanism of acute and chronic liver injury. Gut (2005) 54(7):1024–33. doi: 10.1136/gut.2004.053850 PMC1774601 15951554

[8] Guicciardi ME Malhi H Mott JL Gores GJ. Apoptosis and necrosis in the liver. Compr Physiol (2013) 3(2):977–1010. doi: 10.1002/cphy.c120020 23720337 PMC3867948

[9] Natori S Selzner M Valentino KL Fritz LC Srinivasan A Clavien PA. Apoptosis of sinusoidal endothelial cells occurs during liver preservation injury by a caspase-dependent mechanism. Transplantation (1999) 68(1):89–96. doi: 10.1097/00007890-199907150-00018 10428274

[10] Natori S Higuchi H Contreras P Gores GJ. The caspase inhibitor IDN-6556 prevents caspase activation and apoptosis in sinusoidal endothelial cells during liver preservation injury. Liver Transpl (2003) 9(3):278–84. doi: 10.1053/jlts.2003.50019 12619025

[11] Mueller TH Kienle K Beham A Geissler EK Jauch KW Rentsch M. Caspase 3 inhibition improves survival and reduces early graft injury after ischemia and reperfusion in rat liver transplantation. Transplantation (2004) 78(9):1267–73. doi: 10.1097/01.TP.0000141095.06273.10 15548962

[12] Hoglen NC Anselmo DM Katori M Kaldas M Shen XD Valentino KL. A caspase inhibitor, IDN-6556, ameliorates early hepatic injury in an ex vivo rat model of warm and cold ischemia. Liver Transpl (2007) 13(3):361–6. doi: 10.1002/lt.21016 17318854

[13] Baskin-Bey ES Washburn K Feng S Oltersdorf T Shapiro D Huyghe M. Clinical trial of the pan-caspase inhibitor, IDN-6556, in human liver preservation injury. Am J Transplant (2007) 7(1):218–25. doi: 10.1111/j.1600-6143.2006.01595.x 17227570

[14] Raigani S De Vries RJ Carroll C Chen YW Chang DC Shroff SG. Viability testing of discarded livers with normothermic machine perfusion: Alleviating the organ shortage outweighs the cost. Clin Transplant (2020) 34(11):e14069. doi: 10.1111/ctr.14069 32860634 PMC7944462

[15] Feng S Goodrich NP Bragg-Gresham JL Dykstra DM Punch JD DebRoy MA. Characteristics associated with liver graft failure: the concept of a donor risk index. Am J Transplant (2006) 6(4):783–90. doi: 10.1111/j.1600-6143.2006.01242.x 16539636

[16] van Leeuwen OB de Vries Y Fujiyoshi M Nijsten MWN Ubbink R Pelgrim GJ. Transplantation of high-risk donor livers after ex situ resuscitation and assessment using combined hypo- and normothermic machine perfusion: A prospective clinical trial. Ann Surg (2019) 270(5):906–14. doi: 10.1097/SLA.0000000000003540 31633615

[17] Sanders JA Lakhani A Phornphutkul C Wu KY Gruppuso PA. The effect of rapamycin on DNA synthesis in multiple tissues from late gestation fetal and postnatal rats. Am J Physiol Cell Physiol (2008) 295(2):C406–13. doi: 10.1152/ajpcell.00450.2007 PMC2518428 18550700

[18] Sosa RA Zarrinpar A Rossetti M Lassman CR Naini BV Datta N. Early cytokine signatures of ischemia/reperfusion injury in human orthotopic liver transplantation. JCI Insight (2016) 1(20):e89679. doi: 10.1172/jci.insight.89679 27942590 PMC5135282

[19] Orning P Lien E. Multiple roles of caspase-8 in cell death, inflammation, and innate immunity. J Leukoc Biol (2021) 109(1):121–41. doi: 10.1002/JLB.3MR0420-305R PMC8664275 32531842

[20] Raigani S De Vries RJ Uygun K Yeh H. Pumping new life into old ideas: Preservation and rehabilitation of the liver using ex situ machine perfusion. Artif Organs (2020) 44(2):123–8. doi: 10.1111/aor.13579 31691326

[21] Scheuermann U Zhu M Song M Yerxa J Gao Q Davis RP. Damage-associated molecular patterns induce inflammatory injury during machine preservation of the liver: Potential targets to enhance a promising technology. Liver Transpl (2019) 25(4):610–26. doi: 10.1002/lt.25429 PMC6593678 30734488

[22] Xu D Zhao H Jin M Zhu H Shan B Geng J. Modulating TRADD to restore cellular homeostasis and inhibit apoptosis. Nature (2020) 587(7832):133–8. doi: 10.1038/s41586-020-2757-z 32968279

[23] Mergental H Laing RW Kirkham AJ Perera M Boteon YL Attard J. Transplantation of discarded livers following viability testing with normothermic machine perfusion. Nat Commun (2020) 11(1):2939. doi: 10.1038/s41467-020-16251-3 32546694 PMC7298000

[24] de Vries Y Matton APM Nijsten MWN Werner MJM van den Berg AP de Boer MT. Pretransplant sequential hypo- and normothermic machine perfusion of suboptimal livers donated after circulatory death using a hemoglobin-based oxygen carrier perfusion solution. Am J Transplant (2019) 19(4):1202–11. doi: 10.1111/ajt.15228 PMC6590255 30588774

[25] Martins PN Buchwald JE Mergental H Vargas L Quintini C. The role of normothermic machine perfusion in liver transplantation. Int J Surg (2020) 82S:52–60. doi: 10.1016/j.ijsu.2020.05.026 32417462

[26] Harrison SA Goodman Z Jabbar A Vemulapalli R Younes ZH Freilich B. A randomized, placebo-controlled trial of emricasan in patients with NASH and F1-F3 fibrosis. J Hepatol (2020) 72(5):816–27. doi: 10.1016/j.jhep.2019.11.024 31887369

[27] Kramer G Erdal H Mertens HJ Nap M Mauermann J Steiner G. Differentiation between cell death modes using measurements of different soluble forms of extracellular cytokeratin 18. Cancer Res (2004) 64(5):1751–6. doi: 10.1158/0008-5472.CAN-03-2455 14996736

