# Supplementary figures and images for: Pan-caspase inhibition during normothermic machine perfusion of discarded livers mitigates ex situ innate immune responses
Source: Front Immunol. 2022 Jul 26;13:940094. doi: 10.3389/fimmu.2022.940094 (PMC9360556; doi:10.3389/fimmu.2022.940094)

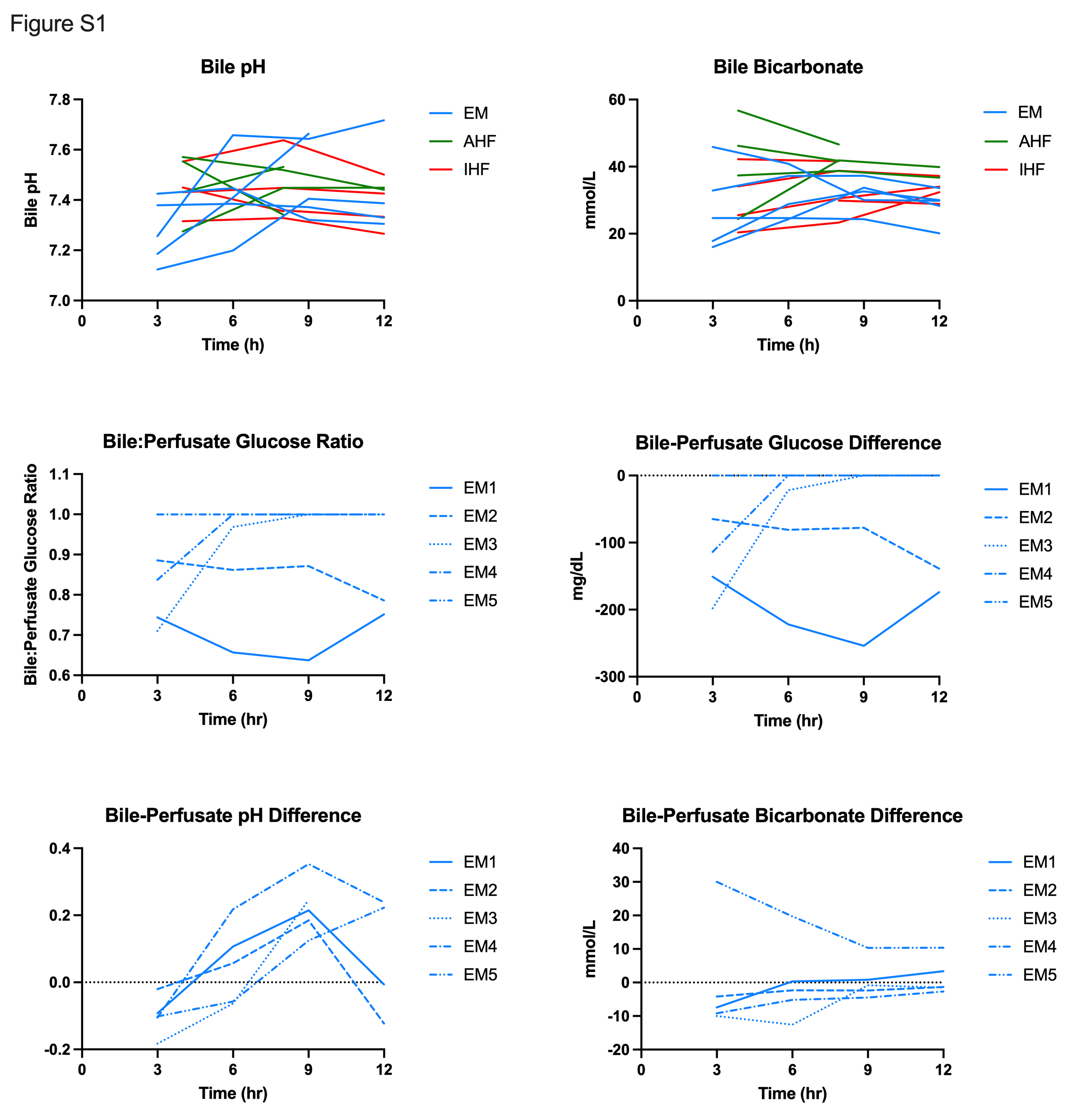

Supplement: Supplementary Figure 1 — Cholangiocellular functional criteria during liver NMP. Various cholangiocellular functional criteria demonstrated. Technical issues with bile collection prevented accurate measurement of bile in two livers from the AHF group. Bile pH and bicarbonate is shown collectively for all available livers. Additional functional data comparing bile and perfusate glucose, pH, and bicarbonate is shown only for EM livers as equivalent data points were not available for the control livers. NMP, normothermic machine perfusion; AHF, adequate hepatocellular function; IHF, inadequate hepatocellular function; EM, emricasan. [file Image_1.tiff]

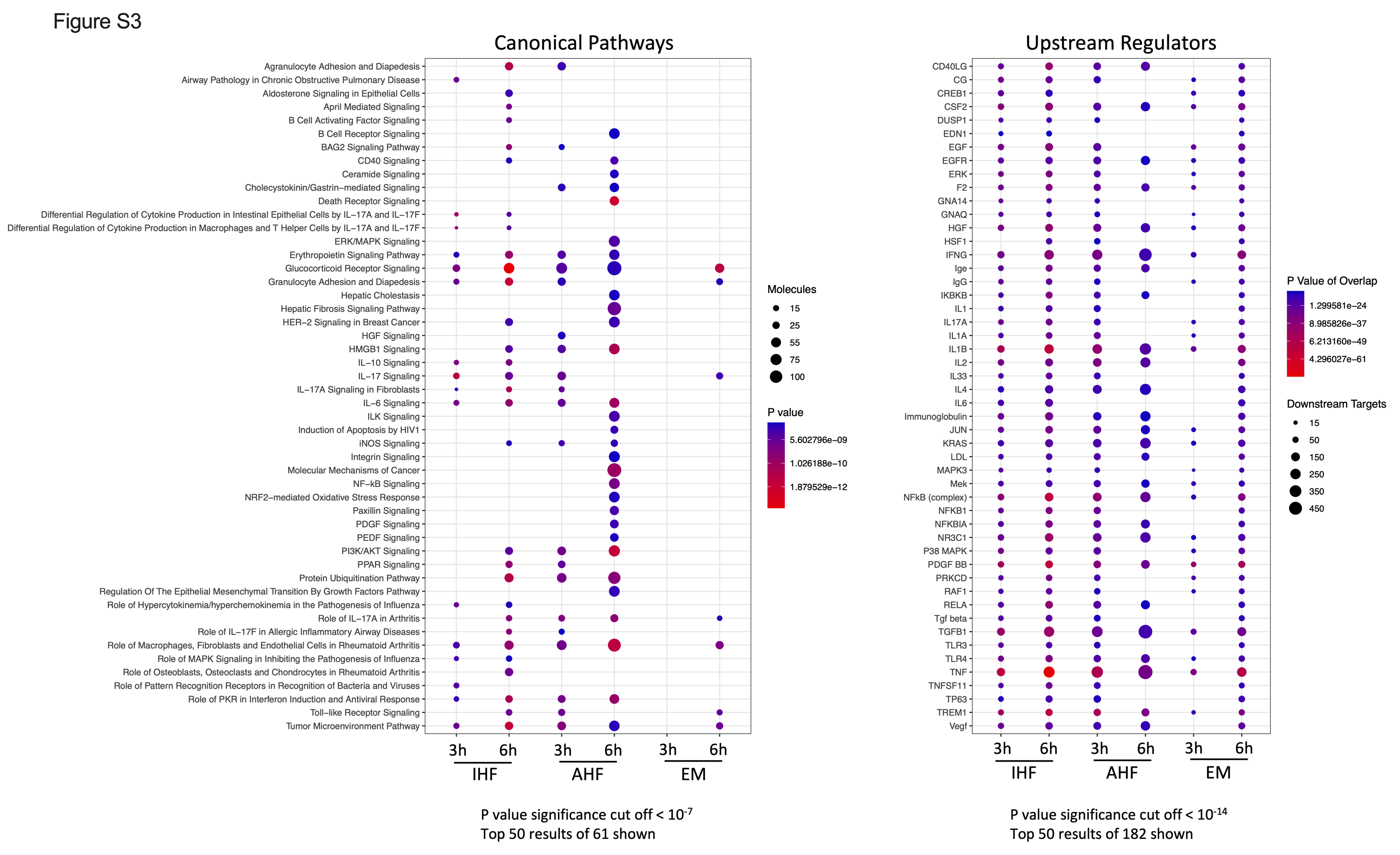

Supplement: Supplementary Figure 3 — Ingenuity Pathway Analysis (IPA) of differentially expressed genes. Dot plots contain significant (A) canonical pathways (CP) and (B) upstream regulators (UR) for the arbitrary P value cut-off indicated in the legend (10-7 for CPs and 10-14 for URs). Results are shown for differentially expressed genes at 3 and 6 hours of NMP compared to the pre-perfusion (0 hour) baseline. The size of each dot represents the number of downstream target genes in our gene set that were in the IPA gene set for that CP or UR. The color of each dot represents the P value for the CP or UR. NMP, normothermic machine perfusion; AHF, adequate hepatocellular function; IHF, inadequate hepatocellular function; EM, emricasan. [file Image_3.tiff]

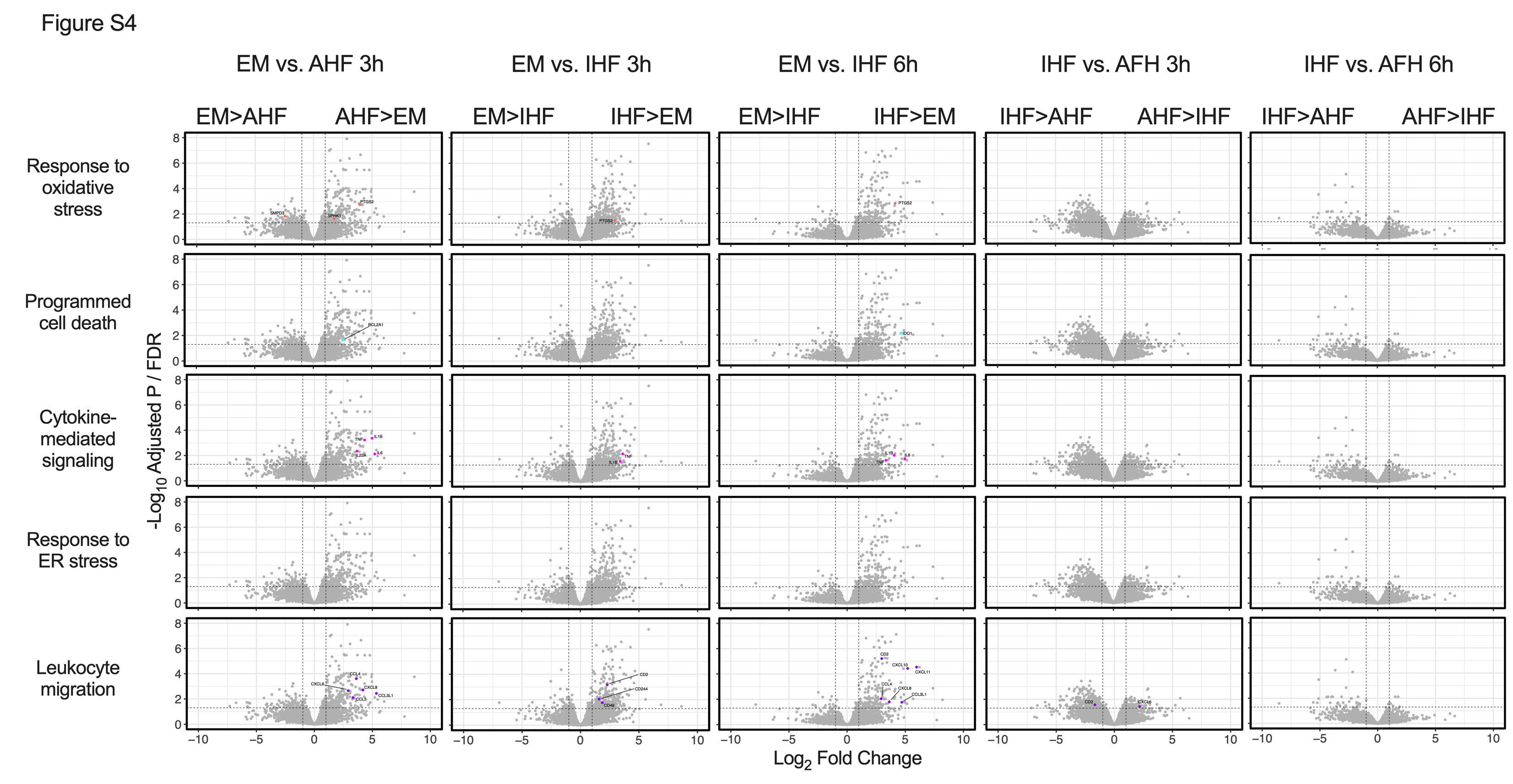

Supplement: Supplementary Figure 4 — Selective volcano plots for all group comparisons. Individual time-point comparisons at 3 and 6 hours of NMP among all groups are demonstrated for the select gene ontology processes demonstrated in . Differentially expressed genes meeting the false discovery rate (FDR) > 0.05 threshold are shown in color and labeled. NMP, normothermic machine perfusion; AHF, adequate hepatocellular function; IHF, inadequate hepatocellular function; EM, emricasan. [file Image_4.tiff]

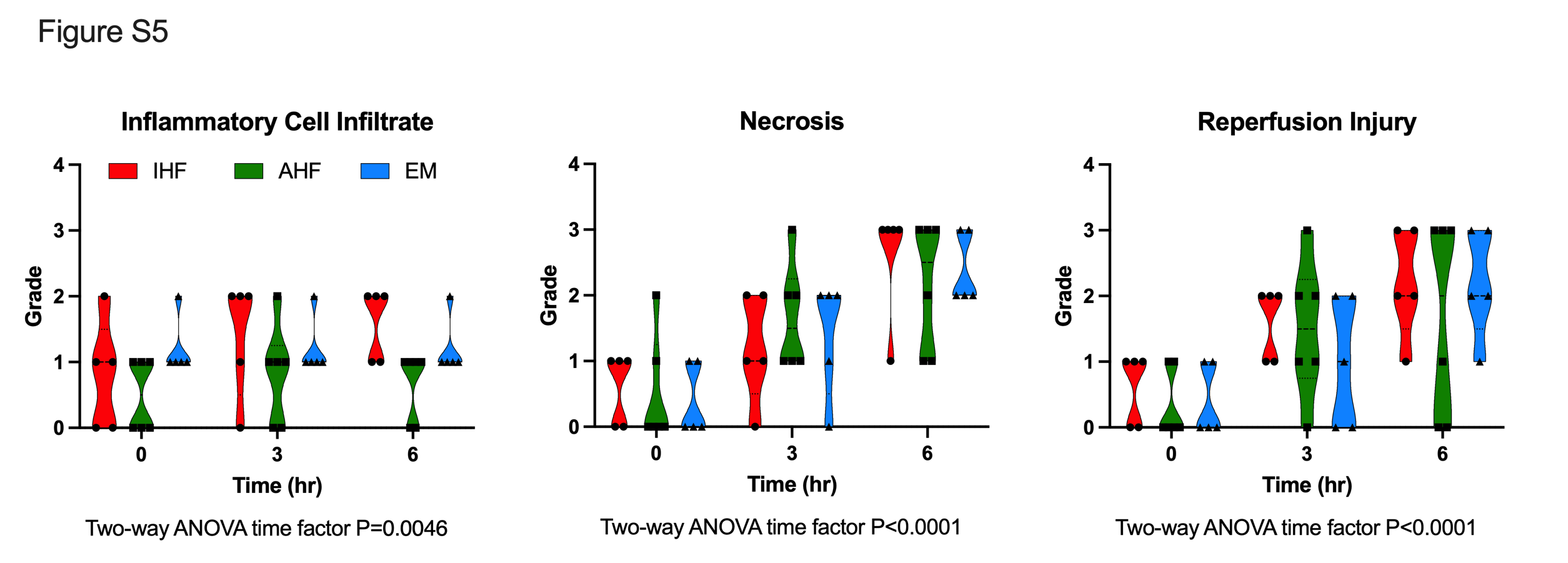

Supplement: Supplementary Figure 5 — Histopathologic reperfusion injury during NMP. Blinded pathologists scored 0, 3, and 6 hours biopsy samples for (A) inflammatory cell infiltrate, (B) necrosis, and (C) reperfusion injury (a composite of A and B). The two-way ANOVA time factor was statistically significant for all three evaluated histologic markers. No significant differences were seen between groups. NMP, normothermic machine perfusion; AHF, adequate hepatocellular function; IHF, inadequate hepatocellular function; EM, emricasan; ANOVA, analysis of variance. [file Image_5.tiff]

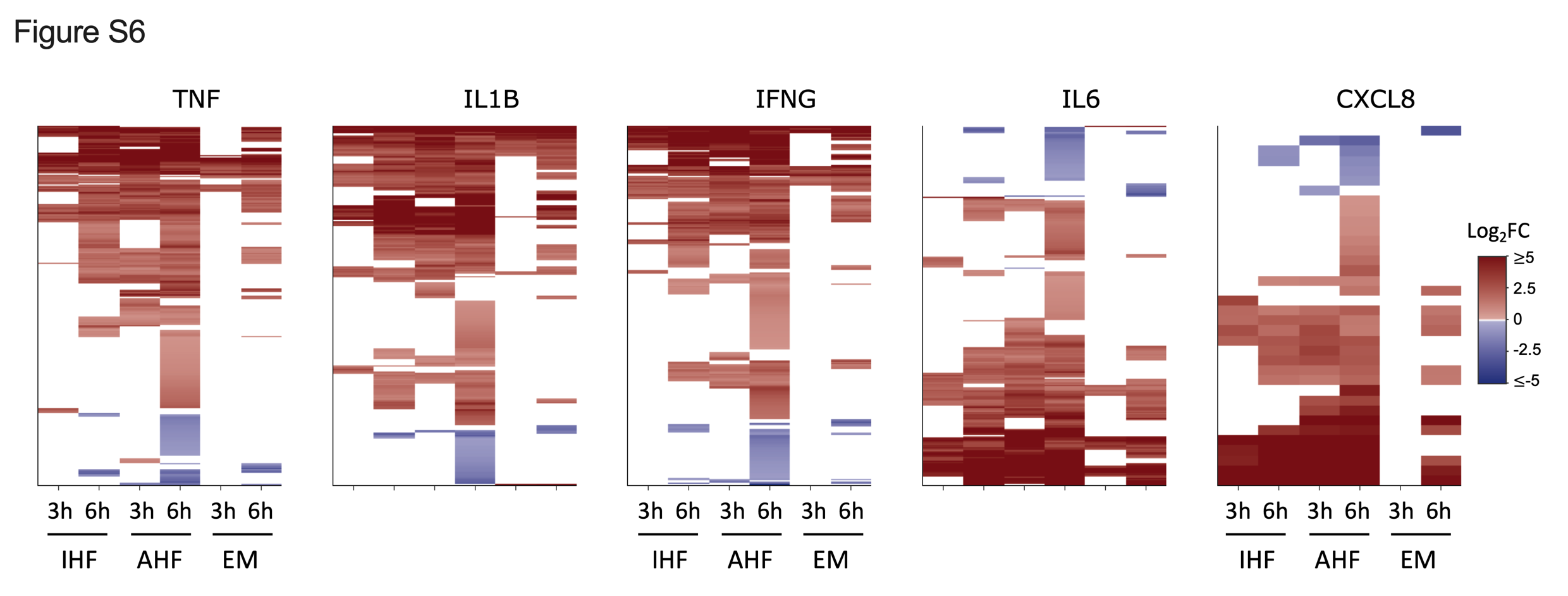

Supplement: Supplementary Figure 6 — Downstream target gene expression for selected proinflammatory cytokine. Proinflammatory cytokines from were analyzed as upstream regulators in IPA for downstream target gene expression. EM livers demonstrate categorically lower enrichment of target genes compared to IHF and AHF groups. NMP, normothermic machine perfusion; AHF, adequate hepatocellular function; IHF, inadequate hepatocellular function; EM, emricasan; IPA, Ingenuity Pathway Analysis; TNF, tumor necrosis factor; IL1B, interleukin-1 β; IFNG, interferon- γ; CXCL8, C-X-C motif chemokine ligand 8 (also known as interleukin-8). [file Image_6.tiff]

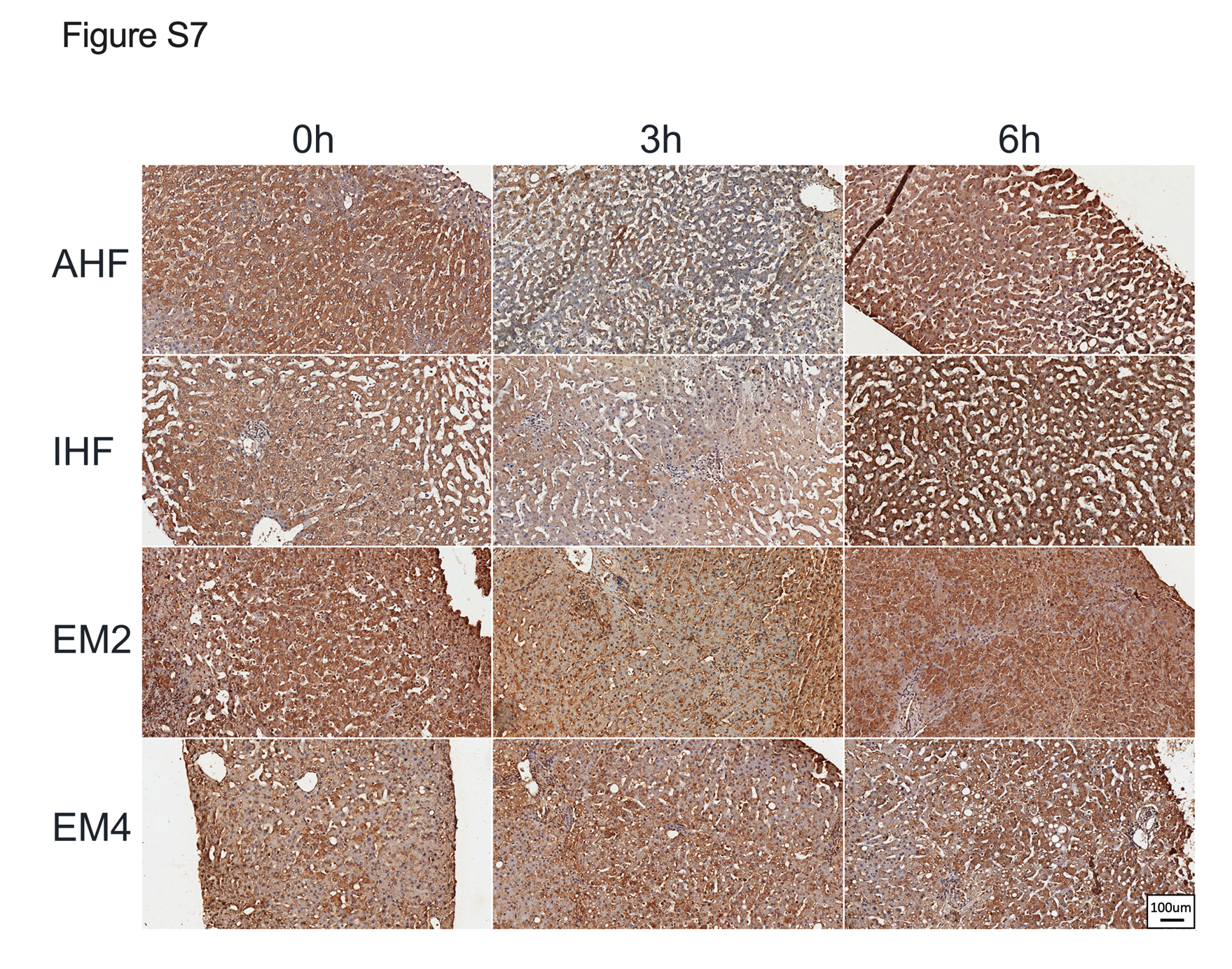

Supplement: Supplementary Figure 7 — LC3B Immunohistochemistry. Immunohistochemistry for LC3B demonstrating patterns of autophagosome assembly during perfusion among the three groups. AHF livers demonstrate active autophagy at 3 and 6 hours of NMP as indicated by the transition from pancytosolic to granular LC3B staining pattern. In contrast, IHF livers demonstrated more sporadic granular LC3B staining after initiation of NMP. EM livers had variable staining with some livers demonstrating granular LC3B staining after 3 hours with return to pancytosolic staining by 6 hours of NMP. 100 micron scale bar shown. NMP, Normothermic machine perfusion; AHF, adequate hepatocellular function; IHF, inadequate hepatocellular function; EM, emricasan; LC3B, microtubule-associated protein 1 light chain 3 beta. [file Image_7.tiff]

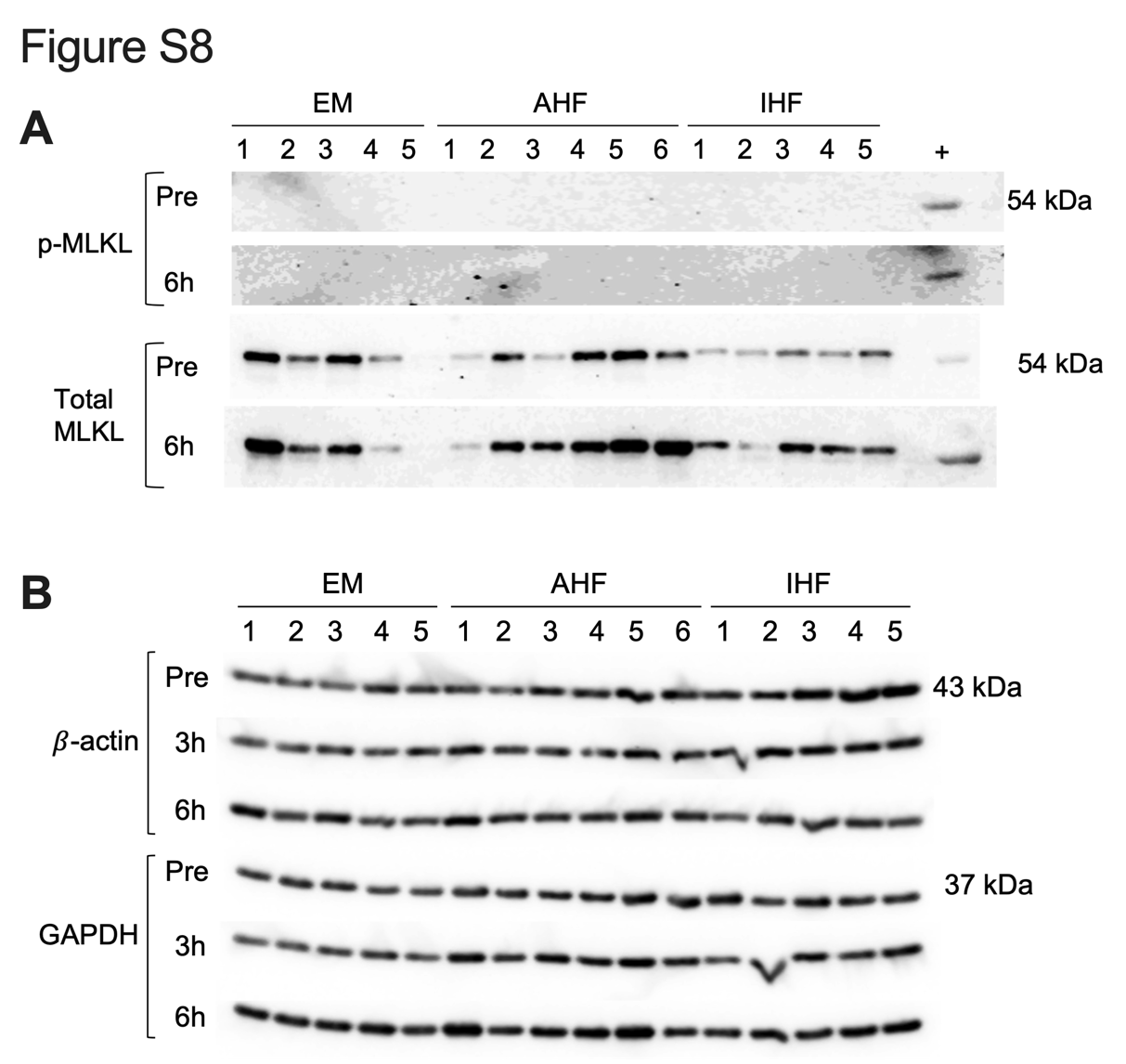

Supplement: Supplementary Figure 8 — Necroptosis and protein controls during NMP. (A) Western immunoblot analysis of phosphorylated and total levels of the necroptosis marker mixed lineage kinase domain-like (MLKL). Phosphorylated MLKL protein was below the level of detection at pre-perfusion and after 6 hours of NMP. (B) β-actin and glyceraldehyde-3-phosphate dehydrogenase (GAPDH) levels for all three groups. NMP, normothermic machine perfusion; AHF, adequate hepatocellular function; IHF, inadequate hepatocellular function; EM, emricasan. [file Image_8.tiff]
