# Supplementary material for: Pan-caspase inhibition during normothermic machine perfusion of discarded livers mitigates ex situ innate immune responses
Source: Front Immunol. 2022 Jul 26;13:940094. doi: 10.3389/fimmu.2022.940094 (PMC9360556; doi:10.3389/fimmu.2022.940094)
Supplement: Supplemental Methods — Detailed description of machine perfusion techniques, RNA sequencing, bioinformatics analysis, protein, and immunohistochemistry studies performed. [file DataSheet_1.docx]

**Pan-caspase inhibition during normothermic machine perfusion of discarded livers mitigates ischemia-reperfusion injury**

**Siavash Raigani^1,3^, John Santiago^2^, Anders Ohman^2^, Megan Heaney^2^, Sofia Baptista^1,3^, Taylor M. Coe^1^, Reinier J. de Vries^3^, Ivy Rosales^4^, Angela Shih^4^, James F. Markmann^1,3^, Philip Gruppuso^2^, Korkut Uygun^1,3^, Jennifer Sanders^2**^, Heidi Yeh^1**^**

^1^Division of Transplant Surgery, Massachusetts General Hospital, Boston, Massachusetts, USA

^2^Department of Pediatrics, Rhode Island Hospital and Brown University, Providence, Rhode Island, USA

^3^Center for Engineering in Medicine and Surgery, Massachusetts General Hospital and Harvard Medical School, Boston, Massachusetts, USA

^4^Department of Pathology, Massachusetts General Hospital, Boston, Massachusetts, USA

** These senior authors contributed equally to this work

Correspondence**:**

Heidi Yeh MD, Division of Transplant Surgery, Department of Surgery, Massachusetts General Hospital. 55 Fruit Street, White 516, Boston, MA, USA. Phone 617-726-3664, Email [hyeh@mgh.harvard.edu](mailto:hyeh@mgh.harvard.edu).

Jennifer Sanders PhD, Division of Pediatric Endocrinology, Department of Pediatrics, Rhode Island Hospital. 55 Claverick St, Room 430, Providence, RI US. Phone 401-444-5802, Email jennifer_sanders@brown.edu.

**Supplemental Methods**

**S1. Human liver perfusions**

Discarded human livers

Human livers were procured in standard fashion through two Organ Procurement Organizations (OPO): New England Donor Services (Waltham, MA, USA) and LiveOnNewYork (New York, NY, USA). In addition, one liver was obtained through the International Institute for the Advancement of Medicine (IIAM, Edison, NJ, USA), which originated from the Gift of Life Donor Program (Philadelphia, PA, USA). Informed consent was obtained from donors by the OPO. The Massachusetts General Hospital and Lifespan Institutional Review Boards, as well as the two OPOs and IIAM, approved this study (No. 2011P001496). No organs were procured from prisoners and no vulnerable populations were included in this study.

Procurement of Grafts

Procurement techniques based on donation after circulatory death followed standard methods. Donor livers were flushed in situ with University of Wisconsin solution. Total warm ischemic time was defined as the period from extubation to cold flush. Functional WIT was defined as the period from asystole to cold flush. Cold ischemic time was defined from cold flush to initiation of machine perfusion. All livers were transported via ground courier. After arrival to the laboratory under static cold storage, livers underwent standard back bench preparation for machine perfusion.

**S2. Total RNA purification, sequencing, and analysis**

RNA Purification and Sequencing

Tissues samples from livers taken immediately prior to perfusion and after 3 and 6 hours of perfusion were used for transcriptome sequencing. Core needle biopsies from the right liver lobe were collected in RNAlater solution (Sigma-Millipore, Waltham, MA, USA) and stored overnight at 4°C. Tissue samples were then removed from solution and stored at -80°C. Total RNA was isolated using the RNeasy Mini Kit (Qiagen, Germantown, MD, USA) according to manufacturer guidelines. 500ng of each sample was sequenced on an Illumina HiSeq 4000 using 9 PCR cycles by GENEWIZ (South Plainfield, NJ, USA). Raw RNA-Seq data files were transferred to the Brown University computing cluster via sFTP and aligned to the human genome build 38 using STAR 2.7.3a (1). While sequencing was performed in two batches, the alignment was performed on all samples simultaneously using the same settings. To quantify any batch effect in our aligned data, we utilized MBatch 1.7.1 (2) to generate a Dispersion Separability Criterion (DSC) value for the pre-treatment (0hr) timepoints from the two batches, resulting in a DSC of 0.197 (p = 0.671). Due to this low batch effect severity score and insignificant p-value, we did not apply a batch correction to our data.

Bioinformatic analysis

Raw read counts were normalized using the Trimmed Mean of M method following removal of low-count genes (fewer than 10 reads in the smallest library). Differential gene expression analysis was conducted in R (3) using edgeR (4). The significance cutoff for differential gene expression was set to a Benjamini-Hochberg false-discovery rate (FDR or q-value) < 0.05. Canonical pathway and upstream regulator enrichment analysis was generated through Ingenuity Pathway Analysis (IPA, Qiagen) (5). IPA categories were considered significant when the P value was below the P values obtained from 5 similarly sized sets of randomly selected genes with a fold-change in expression ≤1 (6). In this study, the threshold for significance was P<10E-7 for canonical pathways and P<10E-14 for upstream regulators.

Principal component analysis (PCA), volcano plots, Venn diagrams, and heatmaps were created using the following R packages: ggplot2 (2D PCA), plot3D/plot3Drgl (3D PCA), EnhancedVolcano (volcano plots), VennDiagram (Venn diagrams), and heatmaply (heatmaps).

Gene ontology (GO) enrichment analysis was performed using the R package GOseq (7). A Benjamini-Hochberg FDR adjusted p-value of < 0.05 was used for determining significant GO term enrichment. The genes with the highest amount of variance explained by principal component 1, 2, or 3 cumulatively totaling to 10% of the total variance explained by the specified PC were further analyzed. The proportion of a gene’s variance explained by a PC was calculated using the squared loading values generated by the prcomp function in R. GO term analysis was performed on these gene subsets and the significantly enriched GO terms in the biological processes category for each PC were summarized in 75% similarity networks generated in Cytoscape (8). For each PC, the enriched GO terms in each hub of their networks can be found in Dataset S1.

Raw sequence data have been deposited in the Gene Expression Omnibus with accession no. GSE202565 and no. GSE165568 (open access).

References

1. Dobin A, Davis CA, Schlesinger F, Drenkow J, Zaleski C, Jha S, et al. STAR: ultrafast universal RNA-seq aligner. Bioinformatics. 2013;29(1):15-21.
2. Akbani R. MBatch: MD Anderson Cancer Center. Available from: <https://bioinformatics.mdanderson.org/public-software/mbatch/>; 2020.
3. Team RC. R: A language and environment for statistical computing. <https://www.R-project.org/>; 2020.
4. Robinson MD, McCarthy DJ, Smyth GK. edgeR: a Bioconductor package for differential expression analysis of digital gene expression data. Bioinformatics 2010;26:139-140.
5. Qiagen. Qiagen Ingenuity Pathway Analysis (IPA). [https://www.qiagenbioinformatics.com/products/ingenuity-pathway-analysis](https://www.qiagenbioinformatics.com/products/ingenuity-pathway-analysis)); 2020.
6. Lamming DW, Demirkan G, Boylan JM, Mihaylova MM, Peng T, Ferreira J, et al. Hepatic signaling by the mechanistic target of rapamycin complex 2 (mTORC2). FASEB J. 2014;28(1):300-15.
7. Young MD, Wakefield MJ, Smyth GK, Oshlack A. Gene ontology analysis for RNA-seq: accounting for selection bias. Genome Biol. 2010;11(2):R14.
8. Shannon P, Markiel A, Ozier O, Baliga NS, Wang JT, Ramage D, et al. Cytoscape: a software environment for integrated models of biomolecular interaction networks. Genome Res. 2003;13(11):2498-504.

S3. **Enzyme-linked immunosorbent assay (ELISA) kits**

| **Protein** | **Manufacturer** | **Product Number** |
| --- | --- | --- |
| Tumor necrosis factor-alpha (TNF$\alpha$) | Sigma-Millipore | RAB0476 |
| Interleukin-6 (IL-6) | Sigma-Millipore | RAB0306 |
| Interferon-gamma (IFN$\gamma$) | Abcam | ab174443 |
| Cleaved cytokeratin 18 | Abcam | ab254515 |
| Caspase 3/7 | Promega | G8091 |
| Cell-free DNA | Sigma-Millipore | 11774425001 |
| Interleukin-8 (IL-8) | Abcam | ab214030 |
| Interleukin-1 Beta (IL-1B) | Sigma-Millipore | RAB0273 |

Abcam (Waltham, MA, USA), Aviva Systems Biology (San Diego, CA, USA), Sigma-Millipore (Waltham, MA, USA).

**S4. Western immunoblot antibodies**

| **Antibody** | **Manufacturer** | **Product Number** |
| --- | --- | --- |
| eIF2α | Santa Cruz Biotechnology | sc-11386 |
| Phospho-eIF2α | Cell Signaling Technology | 9721 |
| p70 S6 Kinase | Cell Signaling Technology | 2708 |
| Phospho-p70 S6 Kinase (Thr389) | Cell Signaling Technology | 9234 |
| LC3B | Cell Signaling Technology | 2775 |
| β-actin | Santa Cruz Biotechnology | sc-47778 |
| GAPDH | Cell Signaling Technology | 5174 |
| P62 | Cell Signaling Technology | 39749 |
| Beclin | Cell Signaling Technology | 3738 |
| MLKL | Cell Signaling Technology | 14993 |
| Phospho-MLKL | Cell Signaling Technology | 91689 |

Cell Signaling Technology (Danvers, MA, USA), Santa Cruz Biotechnology (Dallas, TX, USA), Sigma-Millipore (Waltham, MA, USA).

To compare target protein expression, densitometry quantification was conducted. Blots for β-actin and GAPDH are shown in Figure S6. Both proteins varied across samples and the changes were not consistent between the 2 proteins, with the variation representing the biologic variability in the human liver. As a result, target relative protein was expressed as a ratio of active to inactive protein intensity (or phosphorylated protein to total protein intensity).

**S5. Immunohistochemistry antibodies and methods**

| **Antibody** | **Manufacturer** | **Product Number** |
| --- | --- | --- |
| LC3B | Novus Biologicals (Littleton, CO, USA) | NB100-2220 |

Immunohistochemistry was performed using antibodies directed toward LC3B (see above). Paraffin sections (6 μm) were deparaffinized with xylene and rehydrated with graded ethanol. Antigen retrieval was performed by sub-boiling in 1X-diluted Dako Target Retrieval Solution for 10 min (DakoCytomation, Inc., Carpinteria, CA). Slides were then quenched in 3% H2O2 and blocked in 2.5% Normal Horse Serum (Vector Labs, Burlingame, CA) before overnight incubation in primary antibody (1:200 dilution) at 4°C. Slides were incubated with a horseradish-peroxidase conjugated secondary rabbit antibody (Vector Laboratories) for 1 hr prior to staining with DAB (Vector Laboratories). Slides were counterstained with hematoxylin. Omission of the primary antibody served as a negative control.
